# *IbMYC2* Contributes to Salt and Drought Stress Tolerance via Modulating Anthocyanin Accumulation and ROS-Scavenging System in Sweet Potato

**DOI:** 10.3390/ijms25042096

**Published:** 2024-02-08

**Authors:** Yuanfeng Hu, Hongyuan Zhao, Luyao Xue, Nan Nie, Huan Zhang, Ning Zhao, Shaozhen He, Qingchang Liu, Shaopei Gao, Hong Zhai

**Affiliations:** Key Laboratory of Sweet Potato Biology and Biotechnology, Ministry of Agriculture and Rural Affairs/Beijing Key Laboratory of Crop Genetic Improvement/Laboratory of Crop Heterosis and Utilization, Ministry of Education, College of Agronomy & Biotechnology, China Agricultural University, Beijing 100193, China; huyuanfeng123@163.com (Y.H.); 18888910810@163.com (H.Z.); xueluyao@caas.cn (L.X.); 17810270201@163.com (N.N.); zhanghuan1111@cau.edu.cn (H.Z.); zhaoning2012@cau.edu.cn (N.Z.); sunnynba@cau.edu.cn (S.H.); liuqc@cau.edu.cn (Q.L.)

**Keywords:** sweet potato, *IbMYC2*, salt and drought tolerance, anthocyanin, ROS-scavenging system

## Abstract

Basic helix–loop–helix (bHLH) transcription factors extensively affect various physiological processes in plant metabolism, growth, and abiotic stress. However, the regulation mechanism of bHLH transcription factors in balancing anthocyanin biosynthesis and abiotic stress in sweet potato (*Ipomoea batata* (L.) Lam.) remains unclear. Previously, transcriptome analysis revealed the genes that were differentially expressed among the purple-fleshed sweet potato cultivar ‘Jingshu 6’ and its anthocyanin-rich mutant ‘JS6-5’. Here, we selected one of these potential genes, *IbMYC2*, which belongs to the bHLH transcription factor family, for subsequent analyses. The expression of *IbMYC2* in the JS6-5 storage roots is almost four-fold higher than Jingshu 6 and significantly induced by hydrogen peroxide (H_2_O_2_), methyl jasmonate (MeJA), NaCl, and polyethylene glycol (PEG)6000. Overexpression of *IbMYC2* significantly enhances anthocyanin production and exhibits a certain antioxidant capacity, thereby improving salt and drought tolerance. In contrast, reducing *IbMYC2* expression increases its susceptibility. Our data showed that IbMYC2 could elevate the expression of anthocyanin synthesis pathway genes by binding to *IbCHI* and *IbDFR* promoters. Additionally, overexpressing *IbMYC2* activates genes encoding reactive oxygen species (ROS)-scavenging and proline synthesis enzymes under salt and drought conditions. Taken together, these results demonstrate that the *IbMYC2* gene exercises a significant impact on crop quality and stress resistance.

## 1. Introduction

In nature, abiotic stress severely impacts the physiological processes of plants, leading to oxidative stress responses, damage to cellular components, and metabolic dysfunction, thereby posing a grave threat to plant production and food security [1,2]. In response to abiotic stress, plants are gradually evolving numerous strategies to mitigate these adverse effects, such as active oxygen-scavenging systems, stress-responsive resistance genes, and the production of resistance substances [3,4].

Anthocyanins, as the primary sources of plant stress-related secondary metabolites, exhibit strong antioxidant properties by clearing reactive oxygen species (ROS), minimizing cell damage, maintaining osmotic balance, and enhancing plants’ resistance to abiotic stress [5,6,7]. In recent years, researches on the biosynthetic pathway of anthocyanins have been reported in plants [8,9,10]. As the direct precursor of flavonoid biosynthesis, phenolic compounds undergo catalytic action successively by phenylalanine ammonia lyase (PAL), cinnamate 4-hydroxylase (C4H), and 4-coumarate CoA ligase (4CL) to form 4-coumarate CoA. Then, the 4-coumarate CoA is transformed into dihydroflavonols by chalcone isomerase (CHI), chalcone synthase (CHS), and flavanone 3-hydroxylase (F3H). Finally, anthocyanins are synthesized via dihydroflavonol 4-reductase (DFR) and UDP-glucose-flavonoid 3-O-glucosyltransferase (UFGT) and transported to vacuoles [11,12].

Transcription factors (TFs) can directly regulate the expression of structural genes and downstream TFs associated with anthocyanin biosynthesis, thereby affecting anthocyanin synthesis and metabolism. Among them, the role of the bHLH family in anthocyanin accumulation has been experimentally confirmed. In grapevines, VabHLH137 enhances proanthocyanins and anthocyanin accumulation by directly activating *VaLAR2* expression [13]. In apple, MdbHLH3 functions as a crucial regulator to modulate anthocyanin levels, fruit ripening, and leaf abscission [14]. In addition, a transcriptional complex formed by MYB, bHLH, and WD40-repeat proteins collectively participates in the anthocyanin production pathway [10,15]. In pear, PpbHLH64 interacts with PpMYB10 to stimulate MBW complex formation and activates the expression of *PpUFGT*, thereby significantly enhancing the accumulation of anthocyanins [16]. Co-expression of *PalbHLH1* and *PalMYB90* could enhance antioxidant activities and flavonoid content, further contributing to pathogen resistance [17]. In apple, MdMYB305 interacts with MdbHLH33 to negatively modulate anthocyanin biosynthesis and positively modulate sugar accumulation, while MdMYB10 could interact with MdbHLH33, positively modulating anthocyanin biosynthesis [18].

MYC2, a core TF of the bHLH family, is widely distributed in plant hormone signal transduction [19,20], secondary metabolite synthesis [21], stress response [22,23], and plant growth and development [24,25]. Under salt stress, overexpression of *MYC2-like* improves salt resistance in rice via upregulating *OsCYP2* expression and improving antioxidant activity [26]. AtMYC2 regulates plant drought resistance by raising the transcription of ABA synthesis pathway-related genes *rd22* and *AtADH1* [27]. *MiR1119* and its target gene *MYC2* form a module that regulates wheat’s drought tolerance by alleviating oxidative stress damage [28]. Some studies reveal the influence of *MYC2* in anthocyanin synthesis. Overexpression of *MYC2* increases the content of anthocyanins in transgenic *Arabidopsis thaliana* [29], *Malus pumila* Mill. [30], *Salvia miltiorrhiza* [31], and *Triticum aestivum* L. [32]. Nevertheless, the process of transcriptional regulation in anthocyanin accumulation mediated by MYC2 is still unclear in plants.

Sweet potato (*Ipomoea batata* (L.) Lam.), an essential worldwide food grain, feed grain, and industrial raw material, exerts an irreplaceable influence on agriculture development. The purple-fleshed sweet potato cultivar not only contains abundant anthocyanins but also exhibits strong stress resistance [33,34]. Due to the complex genetic background, incompatible hybridization, and limited germplasm resources of sweet potato, conventional breeding faces many difficulties. Therefore, utilizing genetic engineering technology acts as a contributing approach to rapidly enhancing the quality and stress tolerance of sweet potato [35,36,37,38,39,40,41]. In this research, we demonstrate that overexpressing *IbMYC2* stimulates the anthocyanin pathway in sweet potato. IbMYC2 could combine with the promoter regions of *IbCHI* and *IbDFR* to regulate anthocyanin synthesis-related gene expression. In addition, the overexpression of *IbMYC2* in sweet potato improves salt and drought tolerance by activating the ROS-scavenging system. These results provide an important perspective on how bHLH transcription factors, especially *IbMYC2*, regulate the balance between anthocyanin biosynthesis and abiotic resistance in sweet potato.

## 2. Results

### 2.1. Identification and Sequence Analysis of IbMYC2

**Figure 1:**
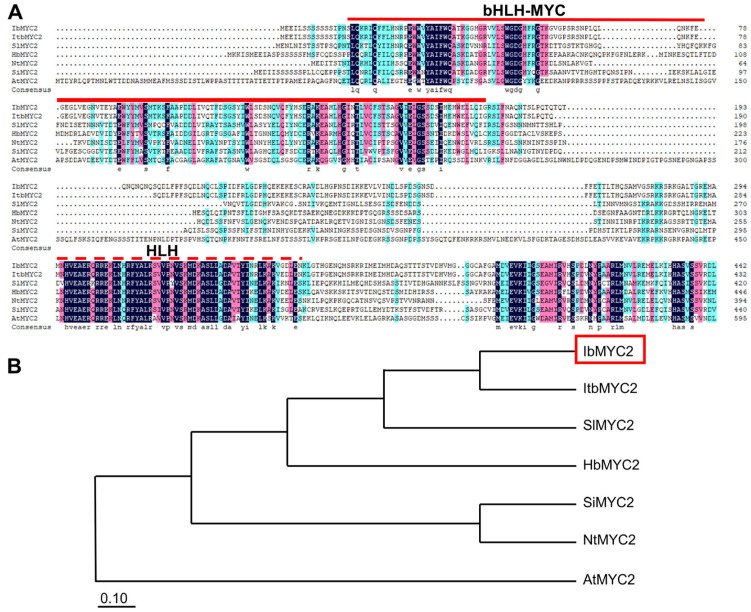
Analyzing the sequence of IbMYC2. (**A**). Alignment of the IbMYC2 sequence with its homolog sequences from other plants, highlighting the conserved amino acids (aa) in distinct colors. The preserved bHLH-MYC and HLH domains are dented by the continuous and discrete red lines, respectively. The similarities of the sequences are represented in black, red, and blue backgrounds. Plant species include *Ipomoea batata* (Ib), *Ipomoea trilobal* (Itb), *Solanum lycopersicum* (Sl), *Sesamum indicum* (Si), *Nicotiana tabacum* (Nt)*, Hevea brasiliensis* (Hb), and *Arabidopsis thaliana* (At). (**B**) Phylogenetic analysis of IbMYC2 from different plants utilizing the neighbor-joining method in MEGA 6.0 with 1000 bootstrap iterations. IbMYC2 is marked using a red box.

We previously obtained the differentially expressed sequence tag (EST) sequence of *IbMYC2* by analyzing the transcriptome data from Jingshu 6 and JS6-5 [42]. Here, the *IbMYC2* was cloned through the homology cloning approach. The open reading frame of *IbMYC2* is 1419 bp, encoding a polypeptide consisting of 472 amino acids (aa). The predicted molecular weight of *IbMYC2* is 53.04 kDa with an isoelectric point (pI) of 6.26. This gene is a member of the bHLH transcription factor family, with a typical bHLH-MYC domain and an HLH domain. The IbMYC2 protein shares a conserved sequence consistent with MYC2 proteins in *Ipomoea triloba* (XP_031095962.1, 96.82%), *Solanum lycopersicum* (XP_004244656.1, 44.54%), *Sesamum indicum* (XP_011081344.1, 44.51%), *Nicotiana tabacum* (XP_016500166.1, 43.88%), *Hevea brasiliensis* (XP_021668275.1, 41.55%), and *Arabidopsis thaliana* (AT1G32640.1, 18.63%) (Figure 1A). IbMYC2 has the closest relationship with ItbMYC2, as shown by phylogenetic analysis (Figure 1B). The genomic sequence of *IbMYC2* is 1419 bp, without introns, and contains only one exon.

### 2.2. IbMYC2 Is Strongly Upregulated in Anthocyanin-Rich Mutant JS6-5 and Responds to Multiple Abiotic Stresses

**Figure 2:**
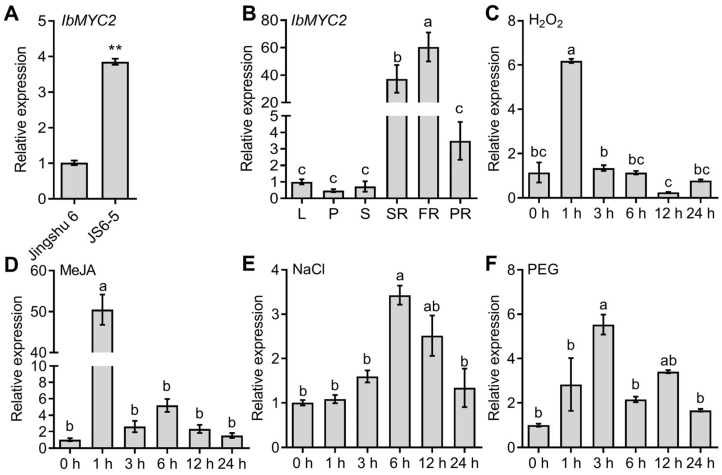
Analysis of *IbMYC2* expression level. (**A**) Analysis of *IbMYC2* mRNA level in the storage roots of Jingshu 6 and JS6-5. The expression in Jingshu 6 was defined as “1”. ** *p* < 0.01; Student’s *t*-test. (**B**) Relative mRNA level of *IbMYC2* across various tissues in JS6-5, including leaves (L), petioles (P), stems (S), storage roots (SR), fibrous roots (FR), and pencil roots (PR). The expression in leaves was defined as “1”. (**C**–**F**) Expression analysis of *IbMYC2* in JS6-5 over various time intervals (h) following exposure to hydrogen peroxide (H_2_O_2_) (100 mmol/L), methyl jasmonate (MeJA) (100 μmol/L), NaCl (200 mmol/L), and polyethylene glycol (PEG)6000 (20%). The expression level at 0 h for each treatment was defined as “1”. As an internal reference, the *ACTIN* gene of sweet potato is utilized. The data are shown as mean values ± SD (*n* = 3). The different lowercase letters denote statistically significant deviations according to one-way ANOVA (Tukey test) at probability levels of *p* < 0.05.

To better verify the potential role of *IbMYC2* in sweet potato, the *IbMYC2* expression level between Jingshu 6 and JS6-5 was analyzed by reverse transcription–quantitative PCR (RT-qPCR). The results showed that *IbMYC2* was strongly upregulated (almost four-fold higher) in JS6-5 than in Jingshu 6 (Figure 2A). In the JS6-5 plants cultivated in the field, *IbMYC2* expression was at the highest level in the root tissue (Figure 2B). Furthermore, under H_2_O_2_, MeJA, NaCl, and PEG6000 conditions, *IbMYC2* expression was strongly upregulated, reaching 6.18-fold (at 1 h), 50.46-fold (at 1 h), 3.42-fold (at 6 h), and 5.53-fold (at 3 h) in JS6-5 plants (Figure 2C–F). These findings suggest that *IbMYC2* might exert an effective influence on anthocyanin accumulation and the abiotic stress response in plants.

### 2.3. IbMYC2 Functions as a Nucleus-Localized Transcriptional Activator

**Figure 3:**
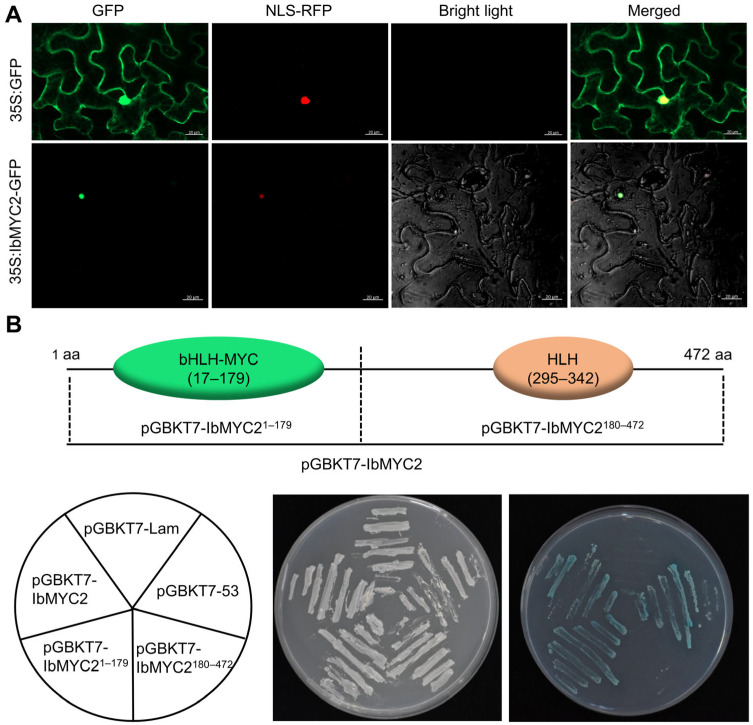
Subcellular localization and transactivation assays of IbMYC2 protein. (**A**) Confocal images of IbMYC2-GFP. IbMYC2-GFP fusion protein is positioned in the nucleus as shown using confocal microscopy in *N. benthamiana* (Bars for 20 μm). (**B**) Transcriptional activation assay of IbMYC2 protein. Fusion proteins, combing the GAL4 DNA-binding domain and various segments of IbMYC2, were expressed in the yeast strain Y2H Gold. The construct of BD-IbMYC2^1–179^ and BD-IbMYC2^180–472^ encompassed IbMYC2 aa 1–179 (containing bHLH-MYC domain) and 180–472 (containing HLH domain) separately. The pGBKT7-53 performed the role of the positive control, while pGBKT7-Lam was the negative control. The BD-IbMYC2, BD-IbMYC2^1–179^, and pGBKT7-53 turned blue when applied to the SD medium lacking His, Ade, and Trp but including X-α-gal.

The entire coding region of *IbMYC2*, excluding the stop codon, was ligated into pCAMBIA1300-GFP, which could produce an IbMYC2-GFP fusion protein. The *35S: IbMYC2-GFP* vector was transiently expressed in epidermal cells of *Nicotiana benthamiana* to exact the subcellular localization. The observation using confocal microscopy displayed that IbMYC2-GFP was positioned in the nucleus, while the GFP protein was distributed throughout the cells, including the cytoplasm, cell membrane, and nucleus (Figure 3A).

To examine whether IbMYC2 harbors transcriptional transactivation function, the full-length IbMYC2 protein was dissected into two fragments, aa 1–179 (containing the bHLH-MYC domain) and 180–472 (containing the HLH domain), and separately inserted into pGBKT7 vector to construct fusion proteins. Then, we transformed these fusion proteins separately into yeast cells. The colonies of yeast carrying either BD-IbMYC2 or BD-IbMYC2^1−179^ turned blue on SD medium lacking His, Ade, and Trp but including X-α-gal (Figure 3B). The above experiments confirm that IbMYC2 functions as a nuclear-localized transcriptional activator, and its N-terminus plays a crucial role in supporting IbMYC2 as a transcriptional activator.

### 2.4. Overexpression of IbMYC2 Improves the Accumulation of Anthocyanin

To explore whether *IbMYC2* influences anthocyanin levels in sweet potato, the *35S: IbMYC2-GFP* vector for overexpression and pFGC5941-*IbMYC2* vector for RNA interference (RNAi) were constructed and individually transfected into Lizixiang embryogenic suspension cells through *Agrobacterium tumefaciens*-mediated transformation (Figure S1). Thirteen independent *IbMYC2*-overexpressed (referred as *IbMYC2*-OE) transgenic plants (OE-1 to OE-13) were identified using genome PCR with 35S-F/*IbMYC2*-ORF-R-specific primers, and three independent *IbMYC2*-RNAi (referred as *IbMYC2*-Ri) transgenic plants (Ri-1 to Ri-3) with pFGC5941-specific primers (Table S1). RT-qPCR analysis was utilized to examine the expression level of *IbMYC2* in the OE and RNAi lines (Figure S1G). Subsequently, two high-expression lines (OE-8 and OE-9) and one low-expression RNAi line (Ri-1) were selected for further study.

**Figure 4:**
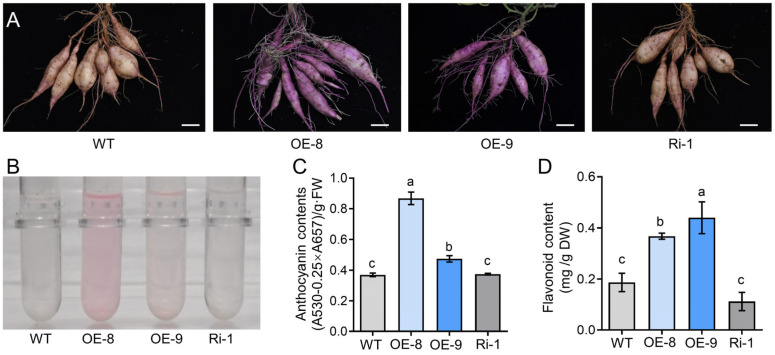
*IbMYC2* contributes to the modulation of anthocyanin accumulation. (**A**) The skin color of the storage roots in *IbMYC2*-OE, *IbMYC2*-Ri, and WT plants after four months’ growth in the field (Bars for 5 cm). (**B**,**C**) The determination of anthocyanin content in the storage roots of *IbMYC2*-OE, *IbMYC2*-Ri, and WT plants. (**D**) The determination of flavonoid content in the storage roots of *IbMYC2*-OE, *IbMYC2*-Ri, and WT plants. The data are shown as mean values ± SD (*n* = 3). The different lowercase letters denote statistically significant deviations according to one-way ANOVA (Tukey test) at probability levels of *p* < 0.05.

*IbMYC2*-OE, *IbMYC2*-Ri, and wild-type (WT) plants were cultured in a greenhouse for acclimation and subsequently transferred to isolated fields for growth (Figure S1H,I). After growing for 4 months, we found that the skin color of the storage roots of *IbMYC2*-OE plants was deep purple, while there was no obvious change in the WT and *IbMYC2*-Ri plants (Figure 4A).

Next, the accumulation of anthocyanins was measured in the storage roots, flesh, and skin separately in *IbMYC2*-OE, *IbMYC2*-Ri, and WT plants. The results revealed that anthocyanin contents in the storage roots, flesh, and skin of *IbMYC2*-OE plants were dramatically higher compared to *IbMYC2*-Ri and WT plants (Figure 4B,C and Figure S2A–D). The storage roots of the *IbMYC2*-OE plants also had higher flavonoid content than the WT plants (Figure 4D). Subsequently, the storage root sections of *IbMYC2*-OE plants, WT plants, and *IbMYC2*-Ri plants were placed at room temperature for 10 min and then subjected to 3,3′-diaminobenzidine (DAB) staining. The staining of the storage root sections of *IbMYC2*-OE plants was lighter compared to WT and *IbMYC2*-Ri plants (Figure S2E). These results indicate that overexpression of *IbMYC2* could enhance the anthocyanin production and also enhance antioxidant capacity in sweet potato.

### 2.5. Genes Covered in Anthocyanin Biosynthesis Are Upregulated in the IbMYC2 Overexpression Plants

**Figure 5:**
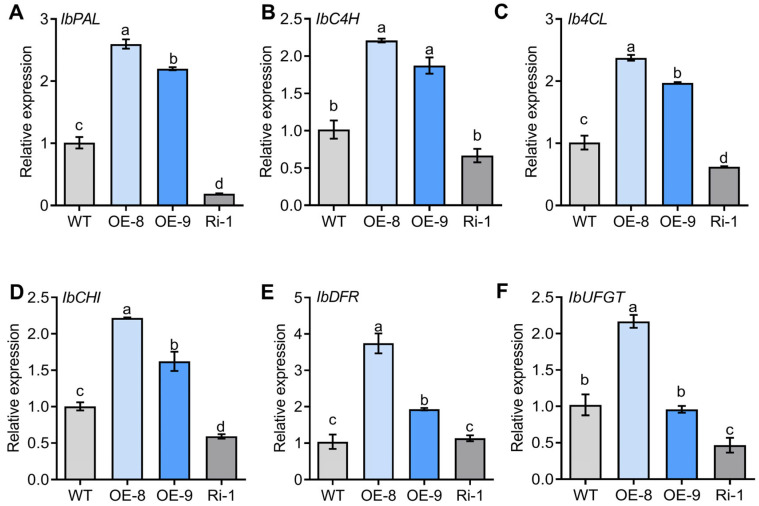
Structural genes related to anthocyanin synthesis are dramatically raised in the *IbMYC2*-OE plants. (**A**–**F**) Expression analysis of genes related to anthocyanin synthesis in the storage roots of plants. The expression level of the storage roots of WT is defined as “1”. The data are shown as mean values ± SD (*n* = 3). The different lowercase letters denote statistically significant deviations according to one-way ANOVA (Tukey test) at probability levels of *p* < 0.05.

To better understand the cause of *IbMYC2* affecting anthocyanin accumulation, the expression level of anthocyanin biosynthesis-related genes was analyzed by RT-qPCR in the storage roots of *IbMYC2*-OE, *IbMYC2*-Ri, and WT plants. The experimental data displayed that the expression levels of *IbPAL*, *IbC4H*, *Ib4CL*, *IbCHI*, *IbDFR*, and *IbUFGT* of *IbMYC2*-OE plants were considerably elevated compared to WT and *IbMYC2*-Ri plants (Figure 5). Further analysis displayed that these genes were expressed more highly in the skin than in the flesh (Figure S3). These data indicate that *IbMYC2* might mainly escalate anthocyanin content by upregulating the expression of structural genes related to anthocyanin synthesis in sweet potato storage roots.

### 2.6. IbMYC2 Can Combine with the Promoter Regions of IbCHI and IbDFR Genes

**Figure 6:**
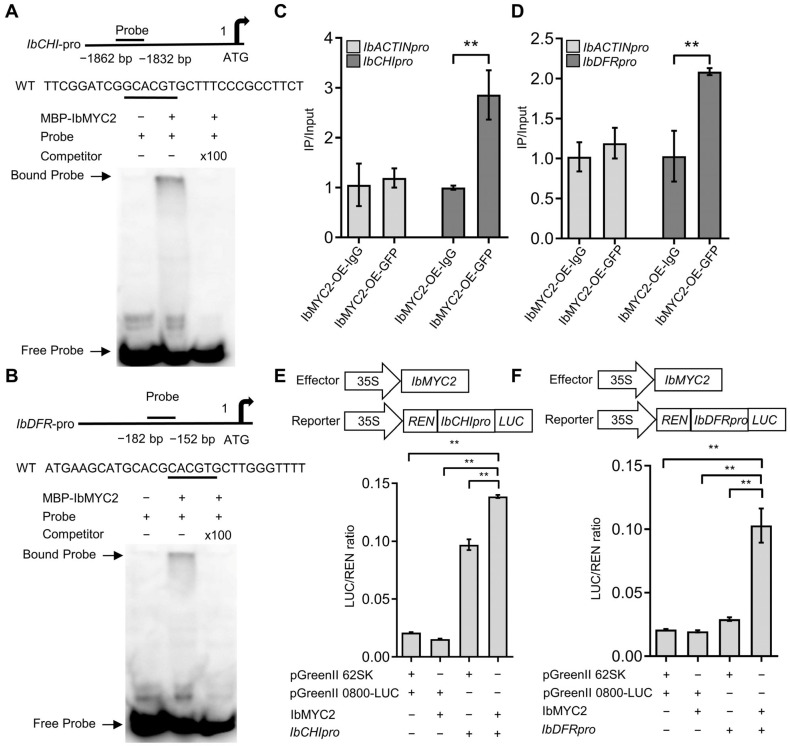
IbMYC2 activates the transcriptional activity of *IbCHI* and *IbDFR*. (**A**,**B**) Electrophoretic mobility shift assays (EMSAs) illustrated the binding of IbMYC2 to the G-box in *IbCHI* and *IbDFR* promoters in vitro. (**C**,**D**) Chromatin immunoprecipitation (ChIP)–qPCR assays, employing 35S: IbMYC2-GFP and 35S: GFP plants applied with anti-GFP antibody, demonstrated the in vivo binding of IbMYC2 to the *IbCHI* and *IbDFR* promoters. Green fluorescent protein is abbreviated as GFP. The control is the *ACTIN* promoter. The data are shown as mean values ± SD (*n* = 3). The symbol ** denotes statistically significant deviations from 35S: GFP at *p* < 0.01 according to Student’s *t*-test. (**E**,**F**) Dual-luciferase (Dual-LUC) assays revealed that IbMYC2 activated the transcriptional activity of *IbCHI* and *IbDFR* in protoplasts. The data are shown as mean values ± SD (*n* = 3). The symbol ** denotes statistically significant deviations at probability levels of *p* < 0.01 according to Student’s *t*-test.

It was discovered that MYC2 could specifically bind to the G-box motif (CACGTG) within the gene promoter, thereby regulating the target gene [43,44]. To better understand how *IbMYC2* regulates anthocyanin accumulation, we discovered G-box elements in the promoter sequences of *IbCHI* and *IbDFR* by searching for differentially expressed gene promoter sequences. In electrophoretic mobility shift assays (EMSAs), MBP-tagged IbMYC2 could be combined with the biotin probes of *IbCHI* and *IbDFR* promoters via G-boxes in vitro (Figure 6A,B). And chromatin immunoprecipitation (ChIP)–qPCR assays further confirmed this in vivo (Figure 6C,D).

To reveal how IbMYC2 modulates these genes’ expression, dual-luciferase (Dual-LUC) assays were performed in sweet potato protoplasts. The results suggested that when *IbCHIpro*-*LUC* was co-transformed with 62SK-*IbMYC2*, *LUC/REN* expression was significantly improved, and a similar result was observed in *IbDFRpro*-*LUC* and 62SK-*IbMYC2*, indicating that IbMYC2 could significantly upregulate the expression of *IbCHI* and *IbDFR* genes (Figure 6E,F). These above results reveal that IbMYC2 could straightforwardly and actively regulate the expression of *IbCHI* and *IbDFR*, and thereby enhance anthocyanin production.

### 2.7. Overexpressing IbMYC2 Improves Salt and Drought Tolerance in Sweet Potato

**Figure 7:**
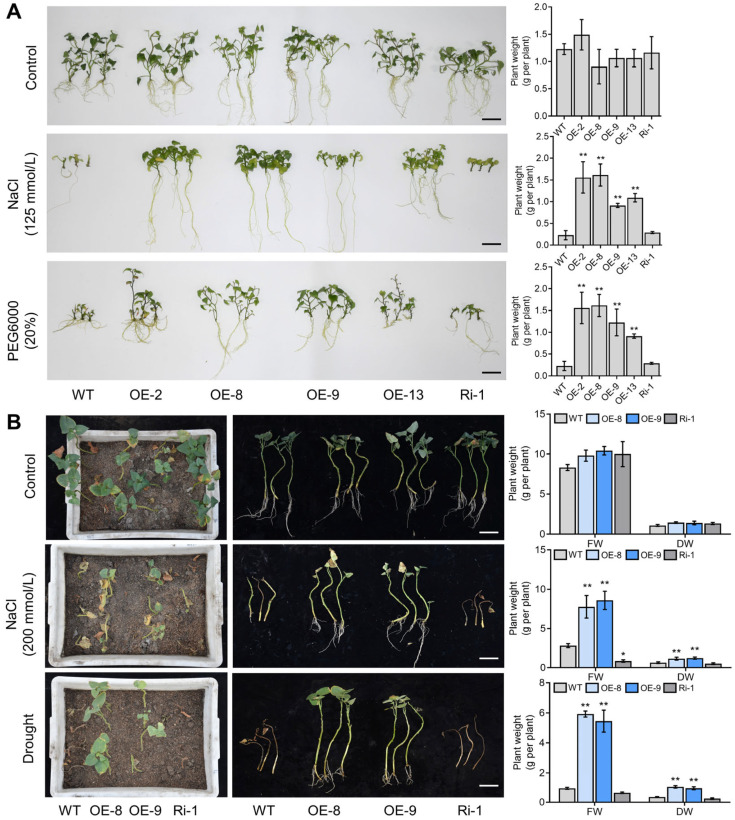
*IbMYC2* positively promotes salt and drought stress tolerance in sweet potato. (**A**) Phenotypes of *IbMYC2*-OE, *IbMYC2*-Ri, and WT plants cultivated on Murashige and Skoog (MS) Medium without stress (control), with NaCl (125 mmol/L), or PEG6000 (20%) treatment for 4 weeks (Bars for 5 cm). (**B**) Phenotypes of *IbMYC2*-OE, *IbMYC2*-Ri, and WT plants grown in transplanting boxes without stress (control), with NaCl (200 mmol/L), or drought treatment (Bars for 10 cm). Fresh weight (FW) and dry weight (DW) are noted. The data are shown as mean values ± SD (*n* = 3). The symbols * and ** denote statistically significant deviations from WT at probability levels of *p* < 0.05 and *p* < 0.01, respectively, according to Student’s *t*-test.

To explore whether *IbMYC2* influences tolerance to salt and drought stress, we cultivated transgenic and WT plants on Murashige and Skoog (MS) medium infused with either 125 mmol/L NaCl or 20% PEG6000 for 4 weeks. Under normal condition, no substantial differences were noted in the growth or physical characteristics of the plants. However, under NaCl or PEG6000 treatment, the stem and leaf development and root formation capabilities of *IbMYC2*-OE plants were markedly enhanced compared to WT plants, while *IbMYC2*-Ri plants showed the opposite changes (Figure 7A; Table S2). Subsequently, stem cuttings from field-grown OE-8, OE-9, Ri-1, and WT plants were cultivated in transplanting boxes and were kept under 200 mmol/L NaCl or drought condition for a six-week duration. Compared to WT plants, *IbMYC2*-OE plants grew better in terms of growth and root development, as well as exhibiting higher fresh weight (FW) and dry weight (DW), while *IbMYC2*-Ri plants died earlier (Figure 7B; Table S3). These findings demonstrated that *IbMYC2* positively promotes salt and drought tolerance in sweet potato.

### 2.8. Genes Encoding ROS-Scavenging and Proline Synthesis Enzymes Are Upregulated in the IbMYC2 Overexpression Plants

**Figure 8:**
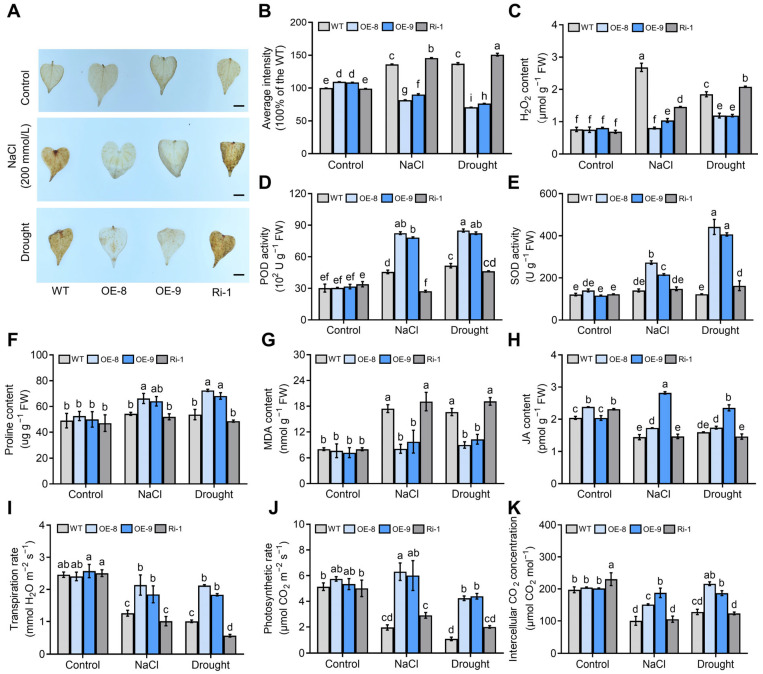
*IbMYC2* stimulates the pathway for reactive oxygen species (ROS) scavenging, photosynthesis, and proline and jasmonic acid (JA) synthesis under both salt and drought conditions in sweet potato. (**A**,**B**) 3,3′-diaminobenzidine (DAB) staining (Bars for 1 cm), (**C**) H_2_O_2_ content, (**D**) activity of peroxidase (POD), (**E**) activity of superoxide dismutase (SOD), (**F**) proline content, (**G**) malondialdehyde (MDA) content, (**H**) JA content, (**I**) transpiration rate, (**J**) photosynthesis rate, and (**K**) intercellular CO_2_ concentration values in the leaves of *IbMYC2*-OE, *IbMYC2*-Ri, and WT plants without stress (control), with NaCl, or drought treatment for 2 weeks. The data are shown as mean values ± SD (*n* = 3). The different lowercase letters denote statistically significant deviations at probability levels of *p* < 0.05 According to two-way ANOVA (Tukey test).

To determine whether ROS-scavenging levels were changed in the transgenic plants, we performed DAB histochemical staining and determination of H_2_O_2_ concentration on the transgenic plants (OE-8, OE-9, and Ri-1) and WT plants subjected to salt or drought stress. Under salt or drought treatment, the *IbMYC2*-OE plants had less intense DAB staining and accumulated less H_2_O_2_, while the *IbMYC2*-Ri plants had more intense DAB staining and produced more H_2_O_2_, compared to WT plants (Figure 8A–C). Furthermore, *IbMYC2*-OE plants demonstrated enhanced activities of peroxidase (POD) and superoxide dismutase (SOD); proline and jasmonic acid (JA) content; and transpiration rate, photosynthetic rate, and intercellular CO_2_ concentration, as well as decreased malondialdehyde (MDA) content. In contrast, the corresponding physiological indices in *IbMYC2*-Ri plants show opposite or no significant differences (Figure 8D–K).

**Figure 9:**
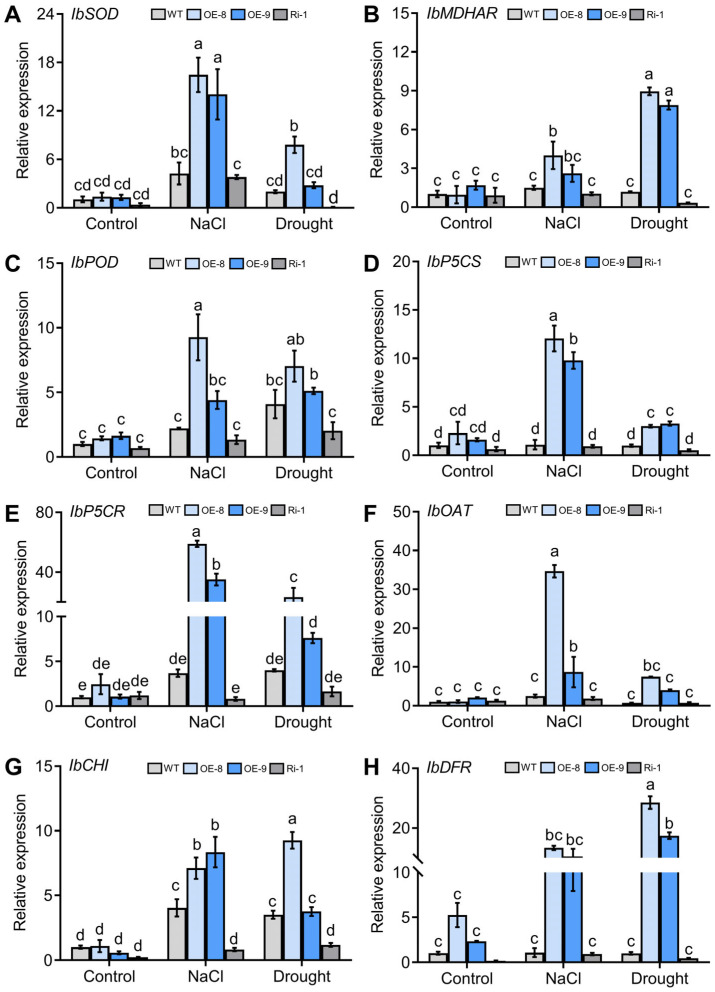
*IbMYC2* positively activates abiotic stress-responsive genes under both salt and drought conditions. Expression levels of (**A**–**C**) ROS scavenging-related gene *IbSOD* (superoxide dismutase gene), *IbMDHAR* (monodehydroascorbate reductase gene), *IbPOD* (peroxidase gene), (**D**–**F**) the proline synthesis-related gene *IbP5CS* (pyrroline-5-carboxylate synthase gene), *IbP5CR* (pyrroline-5-carboxylate reductase gene), and *IbOAT* (ornithine aminotransferase gene) and (**G**,**H**) the anthocyanin biosynthesis-related gene *IbCHI*, *IbDFR* were performed using the leaves of *IbMYC2*-OE, *IbMYC2*-Ri, and WT plants without stress (control), with NaCl, or drought treatment for 2 weeks. The expression level of WT under control is defined as “1”. The data are shown as mean values ± SD (*n* = 3). The different lowercase letters denote statistically significant deviations at probability levels of *p* < 0.05 according to two-way ANOVA (Tukey test).

Further examination showed that under both salt and drought conditions, *IbMYC2* upregulated gene expression concerning the ROS-scavenging system, covering *IbSOD* (superoxide dismutase gene), *IbMDHAR* (monodehydroascorbate reductase gene), *IbPOD* (peroxidase gene), and genes related to proline synthesis, *IbP5CS* (pyrroline-5-carboxylate synthase gene), *IbP5CR* (pyrroline-5-carboxylate reductase gene), and *IbOAT* (ornithine aminotransferase gene) in *IbMYC2*-OE plants. Contrastingly, the above genes were found to be suppressed in *IbMYC2*-Ri plants (Figure 9A–F). In addition, the expression of *IbCHI* and *IbDFR* in *IbMYC2*-OE plants was notably upregulated compared to WT and *IbMYC2*-Ri plants (Figure 9G,H). These findings suggest that *IbMYC2* contributes to the resistance to salt and drought stress, through activating the pathway of ROS scavenging and proline biosynthesis.

## 3. Discussion

The MYC2 transcription factor is instrumental in orchestrating and regulating plant growth and development, hormone interactions, and stress resistance [22,45]. Currently, some reports indicate that the *MYC2* gene performs a positive function in anthocyanin biosynthesis. Under JA treatment conditions, overexpression of *AtMYC2* could not only hinder root growth but also elevate anthocyanin levels in Arabidopsis [29]. Overexpressing *ZmMYC2* also exhibits similar results [46]. In apple, overexpression of *MdMYC2* increases the accumulation of anthocyanins, while inhibition of this gene expression accumulates fewer anthocyanins [30]. *SmMYC2* and *SmbHLH60* can form heterodimers, antagonistically regulating the biosynthesis of anthocyanins in *Salvia miltiorrhiza* [31]. Transient expression of *TsMYC2* is observed to cause anthocyanin improvement specifically in the sheath cells of the leaves [32]. In our research, the *IbMYC2* was cloned from the anthocyanin-rich mutant JS6-5. The IbMYC2 protein has conserved bHLH-MYC and HLH domains (Figure 1A). The phylogenetic tree analysis showed a closer relationship between IbMYC2 and MYC2 in *Ipomoea triloba* (Figure 1B). Additionally, *IbMYC2* expression is predominantly higher in the roots, and it is upregulated by multiple abiotic stresses (Figure 2C–F), suggesting a potential role for IbMYC2 in abiotic stress. Further analysis showed that overexpression of *IbMYC*2 enhances anthocyanin accumulation in sweet potato (Figure 4).

In plants, PAL, CHS, and CHI are important enzymes for forming the precursor substances of flavonoids (including anthocyanins), while DFR, ANS, and UFGT are the key enzymes for converting dihydroflavonols into anthocyanins [8]. During strawberry ripening, the expression of *FaPAL6* is increased along with anthocyanin accumulation [47]. The overexpression of *PdCHS* and *PdCHI* has been shown to significantly increase anthocyanin production in tobacco [48]. In upland cotton, reducing the expression level of *GhDFR1* decreases anthocyanin accumulation, resulting in a lighter coloration of the mature cotton fibers [49]. *VcANS* and *VcUFGT2* have also been confirmed as key players in the final steps of modulating anthocyanin accumulation [50]. In this experiment, compared to WT plants, *IbMYC2* stimulated the expression of *IbPAL*, *IbC4H*, *Ib4CL*, *IbCHI*, *IbDFR*, and *IbUFGT* in *IbMYC2*-OE plants, while these genes were decreased in *IbMYC2*-Ri plants (Figure 5). EMSA, ChIP-qPCR, and Dual-LUC analyses revealed that IbMYC2 could directly and positively regulate *IbCHI* and *IbDFR* genes, thereby causing an increased anthocyanin content in overexpression plants (Figure 6).

The purple-fleshed sweet potato, which contains an abundance of anthocyanins, has received a lot of attention and is recognized as a potential source of valuable natural pigments [6,51]. The flesh color, skin color, and the level of anthocyanin content are three critical characteristics for commodity evaluation and have a significant positive correlation in purple-fleshed sweet potato [52]. However, limited studies have explored the genetic inheritance of these characteristics. In our study, we found that anthocyanin biosynthesis-related genes were notably more highly expressed in the skin than in the flesh (Figure S3), which made *IbMYC2*-OE plants show a deeper skin color and accumulate more anthocyanin content (Figure 4 and Figure S2). These results demonstrate that IbMYC2 participates in the regulation of the skin color of sweet potato, which will contribute to breeding new varieties with elevated anthocyanin content.

Anthocyanins possess certain antioxidant and radical-scavenging abilities, exerting a vital influence on abiotic stress in plants [53]. In Arabidopsis, overexpression of *UGT79B2/B3* can significantly enhance abiotic stress tolerance through modulating anthocyanin abundance and antioxidant activities in plants [54]. RrMYB5 physically interacts with RrMYB10 to increase the accumulation of anthocyanidins by solely or synergistically activating the expression of *RrDFR* and *RrANR*, and eventually enhances plant wounding and oxidative tolerance [55]. *MdNAC104* plays a positive role in modulating apple cold resistance by enhancing anthocyanin synthesis and antioxidant enzyme-encoding gene expression levels [56]. In this study, the color of the storage roots from the *IbMYC2*-OE plants after DAB staining was lighter than that of the WT plants and *IbMYC2*-Ri plants (Figure S2E), indicating less accumulation of ROS. Further assay indicated that the overexpression of *IbMYC2* positively promotes salt and drought tolerance in sweet potato (Figure 7).

**Figure 10:**
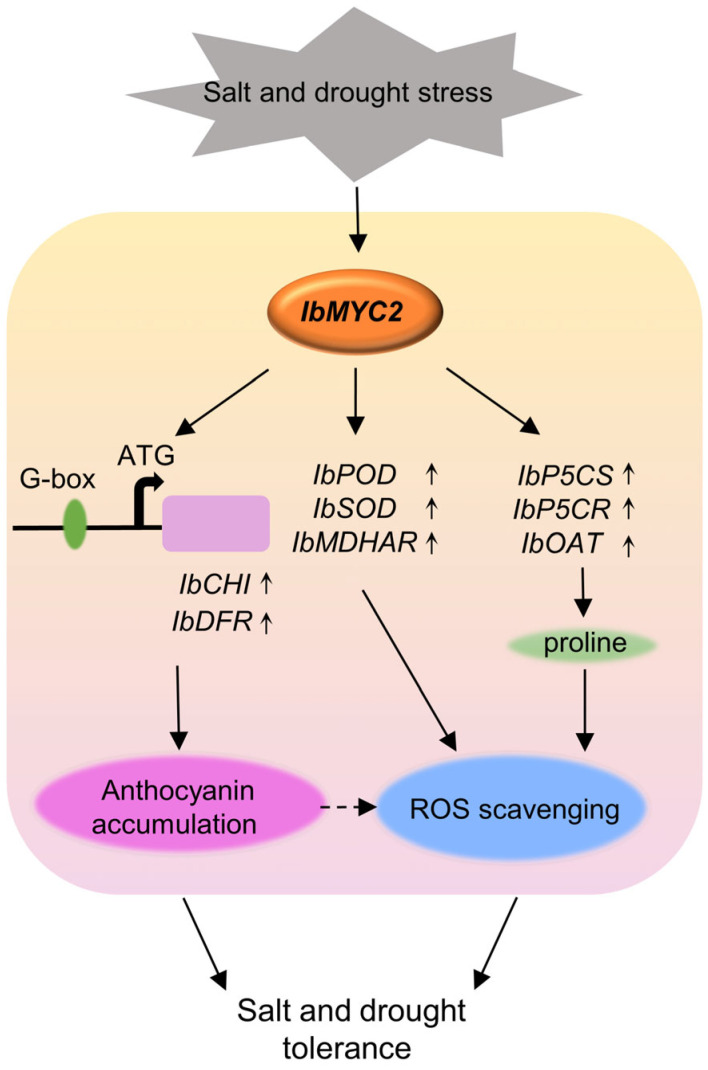
Proposed working model of IbMYC2 in the transgenic sweet potato plants to salt and drought tolerance. 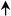 Proposed working model of IbMYC2 in the transgenic sweet potato plants to salt and drought tolerance.

Researches have indicated that structural genes related to anthocyanin synthesis are crucial in affecting plant resistance to abiotic stress. Under cold treatment, overexpression of *IbDFR* enhances the antioxidant properties of anthocyanins by activation of ROS scavenging and thus enhances abiotic tolerance in sweet potato [57]. The *AtDFR* gene could raise anthocyanin accumulation levels, which contributes to salt and drought resistances [58]. In this study, under salt and drought treatment, we observed that *IbCHI* and *IbDFR* expression levels were greatly upregulated in *IbMYC2*-OE plants (Figure 9G,H). In summary, these results suggest that IbMYC2 may enhance the salt and drought tolerance of sweet potato by regulating anthocyanin accumulation and improving antioxidant capacity (Figure 10).

Under abiotic conditions, the accumulation of ROS will markedly escalate in plants, leading to severe oxidative harm to cells, which in turn minimizes enzyme activity and affects photosynthetic apparatus, ultimately impacting plant growth and development [59]. Plants have evolved a complex ROS-scavenging mechanism by activating a series of enzymatic (such as SOD [60], MDHAR [61], and POD [62]) and non-enzymatic antioxidant defenses to maintain cellular ROS homeostasis [63]. Proline also plays a pivotal role in decreasing ROS accumulation and sustaining the redox balance of cells under abiotic stresses [64]. In this study, under salt or drought stress, indices of abiotic stress tolerance were dramatically changed in the *IbMYC2* transgenic plants (Figure 8). The abiotic stress-responsive genes were markedly upregulated in *IbMYC2*-OE plants and downregulated in *IbMYC2*-Ri plants in comparison with WT plants (Figure 9). These findings depict that *IbMYC2* can elevate salt and drought tolerance by the pathway of ROS-scavenging and proline synthesis, thereby protecting plants from damage (Figure 10).

## 4. Materials and Methods

### 4.1. Plant Materials and Growth Conditions

The purple-fleshed sweet potato cultivar Jingshu 6 and its anthocyanin-rich mutant JS6-5 were utilized for cloning and expression of the *IbMYC2*. The pale-yellow-flesh sweet potato cultivar Lizixiang was employed for the functional characterization of *IbMYC2*. Plants grown in vitro were cultivated on MS medium at 27 ± 1 °C and exposed to a light/dark cycle of 13 h of light followed by 11 h of darkness. The transgenic plants were grown in an isolation field, after acclimated in a glasshouse at 25 ± 3 °C for a natural light environment at China Agricultural University, Beijing, China.

### 4.2. Sequence Isolation and Analysis

Extraction of genomic DNA and total RNA were carried out from fresh leaf tissue of JS6-5 plants using EasyPure Plant Genomic DNA Kit (TransGen Biotech, Beijing, China) and TRIzol Reagent (Invitrogen, Carlsbad, CA, USA), respectively. Both the sequences of complementary DNA (cDNA) and genomic DNA were then isolated with primers (*IbMYC2*-ORF-F/R), detailed in Table S1, with the homologous cloning method. INTERPRO database (https://www.ncbi.nlm.nih.gov/Structure/cdd/wrpsb.cgi, accessed on 6 September 2019) was utilized to identify the conserved domains of IbMYC2. A phylogenetic analysis was conducted in MEGA6.0 using the neighbor-joining method with 100 bootstrap iterations [65]. DNAMAN V6 software, provided by Lynnon-BioSoft (San Ramon, CA, USA), was executed to create multiple sequence alignments for IbMYC2 with MYC2 proteins from different plants. Additionally, the SPLIGN program examined the exon–intron structures of IbMYC2 from the website (https://www.ncbi.nlm.nih.gov/sutils/splign, accessed on 17 October 2019).

### 4.3. Expression Analysis of IbMYC2

Total RNA was extracted from the various tissues (including leaf, petiole, stem, storage root, fibrous root, and pencil root) of 3-month-old JS6-5 plants grown in the field. The JS6-5 plants, which were grown in vitro for four weeks, were cultured in half-strength MS medium with added H_2_O_2_ (100 mmol/L), MeJA (100 μmol/L), NaCl (200 mmol/L), and PEG6000 (20%) separately. Sampling of these plants was carried out at various time intervals: 0, 1, 3, 6, 12, and 24 h post-treatment. The experiments were carried out using three biological replicates, each with three plants. To measure transcript abundances, RT-qPCR was employed with SYBR Green Real-Time PCR Master Mix (TaKaRa Biotech, Dalian, China; code: DRR037A) on a 7500 Real-Time PCR instrument (Applied Biosystems, Foster City, CA, USA). The quantification of gene expression was normalized by the sweet potato *ACTIN* (AY905538) gene and calculated utilizing the comparative CT method [66].

### 4.4. Subcellular Localization of IbMYC2

The entire coding region of *IbMYC2*, excluding the stop codon, was ligated into pCAMBIA1300-GFP, which could produce an IbMYC2-GFP fusion protein by the cauliflower mosaic virus (CaMV) 35S promoter. The recombinant vector and the nuclear localization marker (NLS-RFP), the pCAMBIA1300-GFP vector and NLS were separately transiently conveyed into *N. benthamiana* using the *Agrobacterium*-mediated method [67]. After 48 h of cultivation, the observation of fluorescent signals was conducted with a confocal laser-scanning microscope, the LSM880 model from Zeiss (Oberkochen, Germany).

### 4.5. Transactivation Assay in Yeast

The entire region of *IbMYC2*, as well as its segments including amino acids (aa) 1–179 and 180–472, were individually ligated into the pGBKT7 vector (*Nde*I and *Sal*I sites) by homologous recombination. The Yeast Protocols Handbook (Clontech, Mountain View, CA, USA) was utilized to transfer different length fragments of *IbMYC2*, along with pGBKT7-53 (serving as the positive control) and pGBKT7-Lam (the negative control), into yeast strain AH109. SD medium lacking Trp was used to culture transformed yeast colonies. After two days of incubation at 30 °C, the yeast cells were then applied to SD/-Trp/-His/-Ade medium to detect the transcriptional activation.

### 4.6. Transgenic Plant Production

The unique 239 bp sequence of *IbMYC2* was inserted into the RNAi pFGC5941 vector using the method of McGinnis et al. [68]. The *35S: IbMYC2-GFP* and pFGC5941-*IbMYC2* vectors were individually transfected into *A. tumefaciens* strain EHA105. Embryogenic suspension cultures of Lizixiang were utilized for plant transformation and regeneration, adhering to methodologies previously detailed in references [69,70]. To identify the *IbMYC2*-OE and *IbMYC2*-Ri plants, genomic DNA was extracted from the putative transgenic plants for PCR analysis, employing the gene-specific primers listed in Table S1.

### 4.7. Estimation of the Contents of Anthocyanin and Flavonoids

Anthocyanin content was determined using the method of Deikman et al. [71], with minor modifications. The storage roots (0.2 g) were put into 10 mL of extract buffer (methanol/HCl, 99: 1, *v*/*v*), and the sample was extracted overnight and subsequently centrifugated at 5000× *g* for 5 min. A spectrophotometer was utilized to measure the absorbance values (A530 and A657) of the extraction solution. The content of anthocyanin was quantified and expressed as (A530–0.25 × A657)/g fresh weight. Additionally, the content of flavonoids was determined as described in the previous study [72].

### 4.8. Salt and Drought Tolerance Assays

In vitro-grown *IbMYC2*-OE, *IbMYC2*-Ri, and WT plants were exposed to distinct treatments on MS medium enriched with either 125 mmol/L NaCl or 20% PEG6000 during 4 weeks at 27 ± 1 °C with a 13 h light/11 h dark cycle. The FW of the samples was recorded. Cuttings, measuring 25 cm in length, were taken from field-grown *IbMYC2*-OE, *IbMYC2*-Ri, and WT plants. These cuttings were then cultivated in a transplanting box in the glasshouse and watered with half-strength Hoagland solution over two weeks. In the experiments assessing tolerance to salt and drought, four cuttings from each line were subjected to a regimen of irrigation with 300 mL of 200 mmol/L NaCl solution, applied once every three days for four weeks or drought-stressed without watering for eight weeks. Following these treatments, the FW and DW were measured.

Staining with DAB was performed as previously described [73]. The cross-sectional flesh and the leaves were soaked in 1 mg/mL DAB solution overnight and then cleared by ethanol absolute. The activities of POD and SOD enzymes; the content of H_2_O_2_, proline, MDA, and JA; transpiration rate; photosynthesis rate; and intercellular CO_2_ concentration were all measured in the leaves of *IbMYC2*-OE plants, *IbMYC2*-Ri plants, and WT plants by the method described in Zhai et al. [74].

### 4.9. RT-qPCR Analysis of Relative Genes

To verify the anthocyanin synthesis-related gene expression level, the flesh, the skin, and the whole storage roots of *IbMYC2*-OE, *IbMYC2*-Ri, and WT plants were examined using gene-specific primers (Table S1). The leaves of *IbMYC2*-OE, *IbMYC2*-Ri, and WT plants were further utilized to confirm gene expression associated with the ROS-scavenging pathway and proline biosynthesis with gene-specific primers (Table S1). The experiments were carried out using three biological replicates, each with three plants.

### 4.10. EMSA 

The EMSAs were carried out according to the method of Zhang et al. [73]. The coding region of *IbMYC2* was inserted into the pETM-40 vector (*Nco*I and *Xho*lI sites) and further transferred into competent *E. coli* strain Transetta (DE3) cells to obtain the recombinant MBP-IbMYC2 protein. Probes that were labeled with biotin at their 5′ ends served as binding probes, while those without biotin labeling functioned as competitive probes. The specific sequences of these various probes can be discovered in Table S1. Next, the EMSA/Gel-Shift Kit (Beyotime, Shanghai, China) was used for conducting EMSAs referring to the manufacturer’s protocol.

### 4.11. Dual-Luciferase Assay

The promoter regions of *IbDFR* and *IbCHI*, about ~2000 bp upstream of ATG, were inserted into the pGreenII 0800-LUC vector to serve as reporter constructs. And the IbMYC2 coding sequence was inserted into the pGreenII 62-SK vector as an effector. As negative controls, combinations of vectors were utilized: pGreenII 62-SK with either pGreenII 0800-*IbCHIpro* or *IbDFRpro*, pGreenII 62-SK-*IbMYC2* with pGreenII 0800-LUC, and pGreenII 62-SK with pGreenII 0800-LUC. The resultant vectors were transiently co-expressed in sweet potato protoplasts [75]. The dual-luciferase reporter assay system from Promega was applied to quantify levels of Firefly luciferase (LUC) and *Renilla* luciferase (REN) activities. Quantified LUC activity was then standardized against REN activity (Promega, Madison, WI, USA).

### 4.12. ChIP-qPCR Assay

ChIP-qPCR assays were executed following the protocol recorded in Zhang et al. [73]. Briefly, the leaves (approximately 2 g) expressing *35S:IbMYC2-GFP* (OE-8) and *35S:GFP* were treated with 1% (*v*/*v*) formaldehyde, facilitating the cross-linking of protein–DNA complexes. The protein–DNA complexes underwent immunoprecipitation using an anti-GFP antibody (EasyBio, Beijing, China). The DNA from ChIP was purified and subsequently subjected to analysis with qPCR by SYBR mix, employing specific ChIP-qPCR primers as listed in Table S1. To calculate the relative fold enrichment, the target DNA fragment was normalized referring to the values of the respective input subsequently.

### 4.13. Statistical Analysis

Each experiment was replicated biologically three times. All data were analyzed by Student’s *t*-test, one-way ANOVA, or two-way ANOVA with GraphPad Prism 8. The data are shown as mean values ± SD (*n* = 3).

## 5. Conclusions

In summary, the results provide an important perspective on how *IbMYC2* regulates the balance between anthocyanin biosynthesis and abiotic resistance in sweet potato. This study will provide an important gene for sweet potato quality and stress resistance genetic engineering and also offer valuable insights into the mechanism of action of *IbMYC2* in sweet potato and other plants.

## Data Availability

The data presented in this study are available on request from the corresponding author.

## Supplementary Materials

The supporting information can be downloaded at https://www.mdpi.com/article/10.3390/ijms25042096/s1.
