# Supplementary material for: IbMYC2 Contributes to Salt and Drought Stress Tolerance via Modulating Anthocyanin Accumulation and ROS-Scavenging System in Sweet Potato
Source: Int J Mol Sci. 2024 Feb 8;25(4):2096. doi: 10.3390/ijms25042096 (PMC10889443; doi:10.3390/ijms25042096)
Supplement: Supplementary file 1 [file ijms-25-02096-s001.zip › Supplementary_Material.pdf]

Supplementary Material

IbMYC2 Contributes to Salt and Drought Stress Tolerance via Modulating Anthocyanin Accumulation and ROS-Scavenging System in Sweet Potato

Yuanfeng Hu, Hongyuan Zhao, Luyao Xue, Nan Nie, Huan Zhang, Ning Zhao, Shaozhen He, Qingchang Liu, Shaopei Gao* and Hong Zhai*

Key Laboratory of Sweet Potato Biology and Biotechnology, Ministry of Agriculture and Rural Affairs/Beijing Key Laboratory of Crop Genetic Improvement/Laboratory of Crop Heterosis and Utilization, Ministry of Education, College of Agronomy & Biotechnology, China Agricultural University, Beijing 100193, China; huyuanfeng123@163.com (Y.H.); 18888910810@163.com (H.Z.); xueluyao@caas.cn (L.X.); 17810270201@163.com (N.N.); zhanghuan1111@cau.edu.cn (H.Z.); zhaoning2012@cau.edu.cn (N.Z.); sunnynba@cau.edu.cn (S.H.); liuqc@cau.edu.cn (Q.L.)

* Correspondence: spgao@cau.edu.cn (S.G.); zhaihong@cau.edu.cn (H.Z.)

1 Supplementary Figures

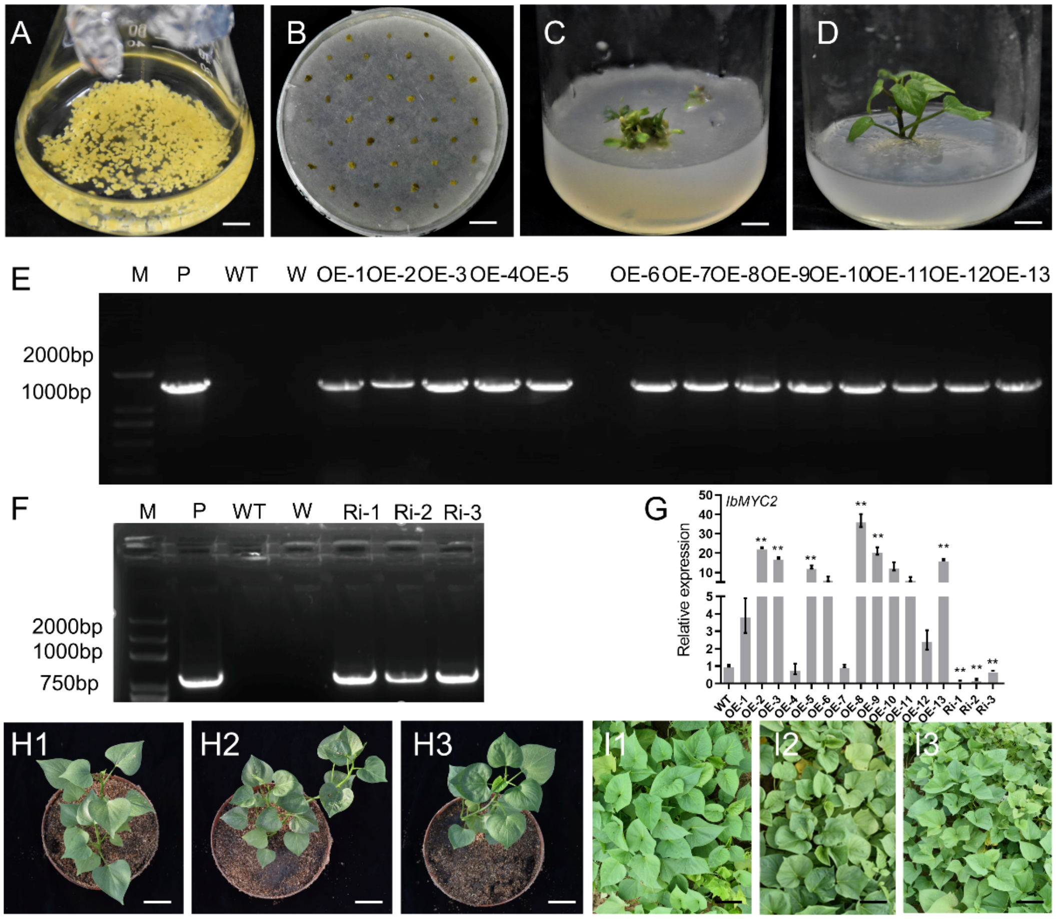

Figure S1. Production of *IbMYC2* transgenic sweet potato plants. (**A**) Proliferation of embryogenic suspension cultures in MS medium containing 2.0 mg/L 2,4-D. Bar = 1 cm. (**B**) Hygromycin (Hyg)-resistant calli (bright yellow) formed after 8 weeks of selection on MS medium containing 2.0 mg/L 2,4-D, 300 mg/L cefotaxime sodium, and 11 mg/L Hyg. Bar = 1 cm. (**C**) Regeneration of plantlets from Hyg-resistant calli on MS medium with 1.0 mg/L abscisic acid (ABA) and 300 mg/L cefotaxime sodium. Bar = 1 cm. (**D**) Transgenic plant cultures on MS medium. Bar = 1 cm. (**E**) PCR analysis of OE transgene-positive plants. Lane M, DL2000 DNA marker; lane P, plasmid pCAMBIA1300-IbMYC2 as a positive control; lane WT, WT as a negative control; lane W, water as a negative control. (**F**) PCR analysis of RNAi transgene-positive plants. Lane M, DL2000 DNA marker; lane P, plasmid pFGC5941-*IbMYC2* as a positive control; lane WT, WT as a negative control; lane W, water as a negative control. (**G**) Relative expression of *IbMYC2* in 4-week-old in vitro–grown transgenic and WT plants. The transcript level of the WT was set to 1.0. The data are shown as mean values ± SD (n = 3). According to Student's t-test, the symbols ** denote statistically dramatically deviations from the wild type (WT), at probability levels of $P < 0.01$. (**H1**-**H3**) The WT, *IbMYC2*-OE, and *IbMYC2*-Ri plants grown for four weeks in the greenhouse. Bar = 10 cm. (**I1**-**I3**) The WT, *IbMYC2*-OE, and *IbMYC2*-Ri plants grown for three months in the isolated field. Bar = 10 cm.

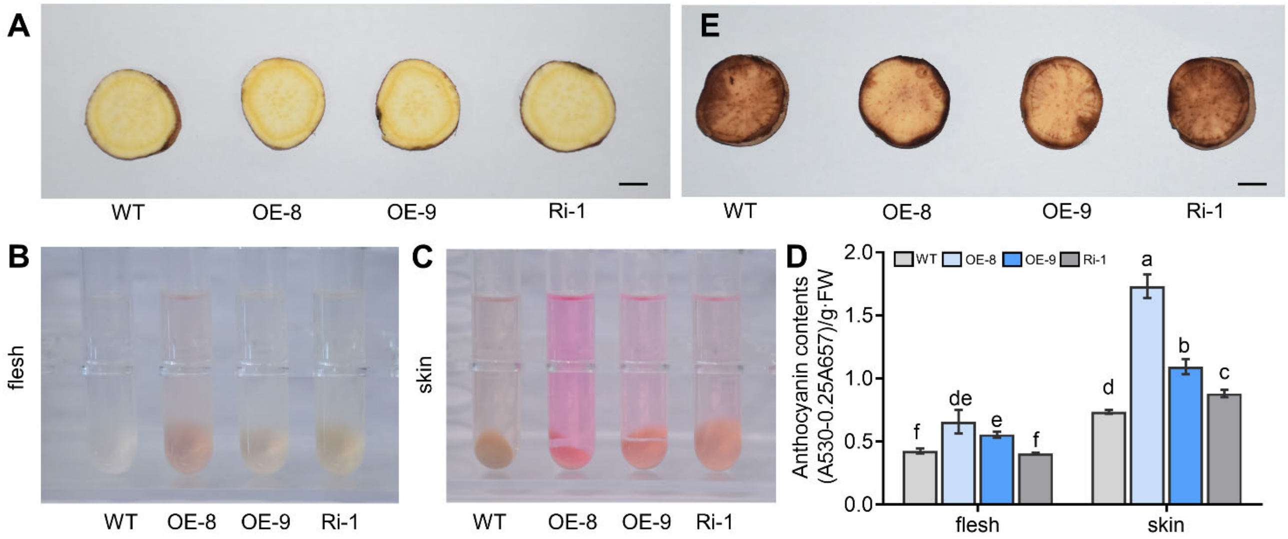

Figure S2. *IbMYC2* regulates anthocyanin accumulation in sweet potato, especially in the skin. (**A**) Storage roots' transverse sections of *IbMYC2*-OE, *IbMYC2*-Ri, and WT plants after four months growth in the field. (Bar for 2 cm). (**B-D**) Anthocyanin contents in the flesh and skin of *IbMYC2*-OE, *IbMYC2*-Ri, and WT plants. (**E**) The DAB staining of storage roots' transverse sections of *IbMYC2*-OE, *IbMYC2*-Ri, and WT plants after being placed at room temperature for 10 minutes. (Bar for 2 cm). The data are shown as mean values ± SD ($n = 3$). According to two-way ANOVA (Tukey test), the different lowercase letters denote statistically dramatic deviations, at probability levels of $P < 0.05$.

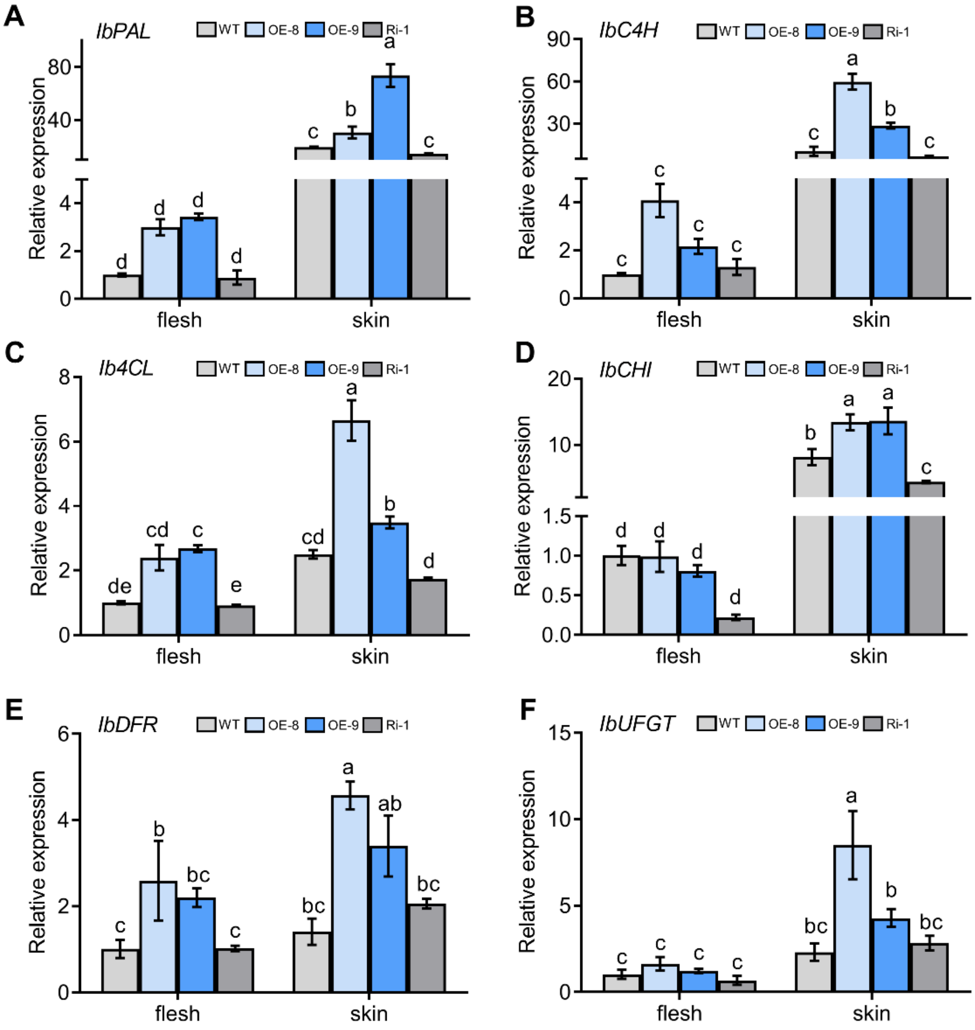

Figure S3. Overexpression of *IbMYC2* up-regulated anthocyanin biosynthesis-related genes in the flesh and skin of sweet potato. (**A**-**F**) Relative expression levels of the anthocyanin biosynthesis-related gene between the flesh and the skin were examined in the storage roots of *IbMYC2* transgenic and WT plants. The expression level of WT in the flesh is defined as "1". The data are shown as mean values ± SD (n = 3). According to two-way ANOVA (Tukey test), the different lowercase letters denote statistically dramatic deviations, at probability levels of $P < 0.05$.
