# Supplementary material for: IbMYC2 Contributes to Salt and Drought Stress Tolerance via Modulating Anthocyanin Accumulation and ROS-Scavenging System in Sweet Potato
Source: Int J Mol Sci. 2024 Feb 8;25(4):2096. doi: 10.3390/ijms25042096 (PMC10889443; doi:10.3390/ijms25042096)
Supplement: Supplementary file 1 [file ijms-25-02096-s001.zip › Table S1.pdf]

Table S1 Sequences of the primers used in this study.

**Primer name**	
**Primers for ORF/genomic DNA**	
IbMYC2 -ORF-F	ATGGAAGAGATTCTTTCTTCATCTT
IbMYC2 -ORF-R	TTAAGACTGCAATCTTCTAAGG
**Primers for subcellular localization and overexpression vectors**	
IbMYC2 -OE-F	GGGGTACCATGGAAGAGATTCTTTCTTCATCTT
IbMYC2 -OE-R	ACGCGTCGACAGACTGCAATCTTCTAAGGATGAC
**Primers for RNAi vectors**	
IbMYC2 -RNAi-F1	CCGCTCGAGGACCACGTGATGGGCGG
IbMYC2 -RNAi-R1	ATTTAAATTCTTCCTCATTGGTCAACCCG
IbMYC2 -RNAi-F2	CGGGATCCTCTTCCTCATTGGTCAACCCG
IbMYC2 -RNAi-R2	GCTCTAGAGACCACGTGATGGGCGG
**Primers for identifying overexpression plants**	
35S-F	GACGCACAATCCCACTATCC
IbMYC2 -ORF-R	TTAAGACTGCAATCTTCTAAGG
**Primers for identifying RNAi transgenic plants**	
pFGC5941F-R	CTACCTTCCCACAATTCGTC
pFGC5941R-F	TACTTACACTTGCCTTGGAG
**Primers for transcriptional activation activity**	
pGBKT7-*IbMYC2* -F	GGAATTCCATATGATGGAAGAGATTCTTTCTTCATCTT
pGBKT7-*IbMYC2* -R	ACGCGTCGACTTAAGCCTGCAATCTTCTAAGG
pGBKT7-*IbMYC2* $^{1\text{-}179}$-F	GGAATTCCATATGATGGAAGAGATTCTTTCTTCATCTT
pGBKT7-*IbMYC2* $^{1\text{-}179}$-R	ACGCGTCGACTTAAAAGATAGAACGCCCGATT
pGBKT7-*IbMYC2* $^{180\text{-}472}$-F	GGAATTCCATATGAACGCACAAAACACTAGCCTC
**Primers for EMSAs**	
pETM-40-*IbMYC2* -F	CATGCCATGGACATGGAAGAGATTCTTTCTTCATC
pETM-40-*IbMYC2* -R	CCGCTCGAGTTAAGCCTGCAATCTTCTAAGG
IbCHI- probe	TTCGGATCGGCACGTGCTTTCCCGCCTTCT
IbDFR -probe	ATGAAGCATGCACGCACGTGCTTGGGTTTT
**Primers for luciferase assays**	
pGreenII-62SK-*IbMYC2* -F	GTGGATCCCCCGGGCTGCAGATGGAAGAGATTCTTTCTTCATCTT
pGreenII-62SK-*IbMYC2* -R	GATTTCAGCGAATTGGTACCTTAAGACTGCAATCTTCTAAGG
pGreenII-0800-*IbCHIpro* -F	TATAGGGCGAATTGGGTACCTTACTACATGAACCACGTTCCACA
pGreenII-0800-*IbCHIpro* -R	GTGGATCCCCCGGGCTGCAGTTCAGTACCCACTGCATATATAAAA
pGreenII-0800-*IbDFRpro* -F	TATAGGGCGAATTGGGTACCGGAAAGATTAGAATGATTTGGGATG
pGreenII-0800-*IbDFRpro* -R	GTGGATCCCCCGGGCTGCAGTATATTTTTTGCTCAGATCAATAAG
**Primers for ChIP-qPCR assays**	
ACTINpro -qPCR-F	CCAACCATCAAAGATTATACGGAGTA
ACTINpro -qPCR-R	GCCACGGTGTCACATTTACGATTT
IbCHIpro -qPCR-F	TTATTGATGCAATGCGCCGGTCTG
IbCHIpro -qPCR-R	AACCTGGACTTGTGGTGCTGGTTG
IbDFRpro -qPCR-F	TATTACCCATTAAGCGTTGAAGTG
IbDFRpro -qPCR-R	TAGATAGGCTCAAAACCCAAGCAC
**Primers for real-time quantitative PCR**	
ACTIN -F	AGCAGCATGAAGATTAAGGTTGTAGCAC
ACTIN- R	TGGAAAATTAGAAGCACTTCCTGTGAAC
IbMYC2 -F	CCATACATGAGCACTGGGAATTG
IbMYC2 -R	AGGGGAAAGACATTGGTTAAGATCT
IbPAL -F	CCCTGCAGTGCTAACTACCC
IbPAL -R	GAATAGCCGGGTTCCCACTC
IbC4H -F	GGTCGTAGTGTCGAACCCTG
IbC4H -R	TGAACACCATGTCCTGACCG
Ib4CL -F	TGGGTTCCACCGGAAAAACA
Ib4CL -R	CCGATCATAGAAGCCGCCAT
IbCHI -F	CCGATCATAGAAGCCGCCAT
IbCHI -R	CTCCGGCATTGAACCCTCTT
IbDFR -F	GATGAAGCCATTCAAGGCTG

IbDFR -R	GCAGTGATTAAGCTAGGTGG
IbUFGT -F	CAAACGGAAACGGGTTGGAC
IbUFGT -R	CGGTGGGTTCTAGCTTCTGG
IbSOD -F	TCCTGGACCTCATGGATTTC
IbSOD -R	GCCACTATGTTTCCCAGGTC
IbMDHAR -F	CTACTCCCGTGCCTTTGATT
IbMDHAR -R	CTCCAAGAATGCACCAACAA
IbPOD -F	TTCACGACTGCTTCGTTGA
IbPOD -R	TTCTCAACCGCGGTCTTAA
IbP5CS -F	GCCTGATGCACTTGTTCAGA
IbP5CS -R	TTGAGCAATTCAGGGACCTC
IbP5CR -F	ATAGAGGCATTGGCTGATGG
IbP5CR -R	GGTAGTCCCACCTGGTGATG
IbOAT -F	ATCCATGAGCTGTGACAACG
IbOAT -R	CTCTCCTTGGATGGGTTCAA
