# Supplementary material for: IbMYC2 Contributes to Salt and Drought Stress Tolerance via Modulating Anthocyanin Accumulation and ROS-Scavenging System in Sweet Potato
Source: Int J Mol Sci. 2024 Feb 8;25(4):2096. doi: 10.3390/ijms25042096 (PMC10889443; doi:10.3390/ijms25042096)
Supplement: Supplementary file 1 [file ijms-25-02096-s001.zip › Table S2.pdf]

Table S2 Comparison of *IbMYC2* -OE and *IbMYC2* -Ri plants with WT plants after 4 weeks of growth on MS medium without stress (control), with NaCl (125 mmol/L), or with PEG6000 (20%) treatment

Plant lines	Fresh weight (g plant^{-1})			Root length (cm)		
	Control	NaCl	PEG6000	Control	NaCl	PEG6000
WT	1.228±0.100	0.230±0.106	0.230±0.106	7.233±1.343	1.567±0.586	5.133±0.351
OE-2	1.492±0.279	1.558±0.359**	1.558±0.359**	13.300±3.961	17.230±3.256**	6.933±0.666**
OE-8	0.906±0.316	1.615±0.254**	1.615±0.254**	9.433±5.084	22.300±1.375**	15.200±2.646
OE-9	1.064±0.160	0.845±0.148*	0.845±0.148*	10.930±0.611	22.970±1.041**	7.600±1.249
OE-13	1.063±0.170	1.225+0.306**	1.225±0.306**	12.730±2.572	10.830±2.466**	2.333±0.757
Ri-1	1.162±0.295	0.290±0.023	0.290±0.023	14.600±4.045	0±0.000	13.870±2.380**

The data are presented as means ± SDs (n = 3). According to Student's t -test, the symbols * and ** indicate a significant difference compared with the WT at $P < 0.05$ and $P < 0.01$, respectively.
