# Supplementary material for: IbMYC2 Contributes to Salt and Drought Stress Tolerance via Modulating Anthocyanin Accumulation and ROS-Scavenging System in Sweet Potato
Source: Int J Mol Sci. 2024 Feb 8;25(4):2096. doi: 10.3390/ijms25042096 (PMC10889443; doi:10.3390/ijms25042096)
Supplement: Supplementary file 1 [file ijms-25-02096-s001.zip › Table S3.pdf]

Table S3 Comparison of *IbMYC2* -OE and *IbMYC2* -Ri plants with WT plants after half-Hoagland solution containing 200 mmol/L NaCl, drought or no stress

	Fresh weight (g plant^{-1})			Dry weight (g plant^{-1})			Root length (cm)		
Plant lines	Control	NaCl	PEG6000	Control	NaCl	PEG6000	Control	NaCl	PEG6000
WT	8.327±0.375	2.813±0.253	0.957±0.068	1.100±0.113	0.653±0.108	0.363±0.025	12.900±2.910	7.450±1.805	1.000±1.732
OE-8	9.807±0.686	7.775±1.435**	5.930±0.197**	1.453±0.102	1.165±0.188**	1.057±0.080**	15.830±1.002	25.480±7.732**	8.600±0.458*
OE-9	10.420±0.529	8.595±1.157**	5.457±0.736**	1.403±0.225	1.233±0.137**	0.963±0.102**	16.330±0.902	24.130±8.141**	11.630±5.160**
Ri-1	10.000±1.556	0.863±0.158*	0.653±0.051	1.340±0.131	0.533±0.099	0.253±0.049	13.930±3.262	4.967±1.595	0±0.000

The data are presented as means ± SDs ($n = 3$). According to Student's *t* -test, the symbols * and ** indicate a significant difference compared with the WT at $P < 0.05$ and $P < 0.01$, respectively.
